# Hypertriglyceridemia impact on arterial parameters in patients with metabolic syndrome

**DOI:** 10.1186/s12872-021-02202-3

**Published:** 2021-08-13

**Authors:** Egidija Rinkūnienė, Vilma Dženkevičiūtė, Žaneta Petrulionienė, Eglė Majauskienė, Ligita Ryliškytė, Roma Puronaitė, Jolita Badarienė, Rokas Navickas, Aleksandras Laucevičius

**Affiliations:** 1 Faculty of Medicine, Vilnius University Hospital Santaros Klinikos, Vilnius University, Santariskiu Str. 2, 08661 Vilnius, Lithuania; 2 State Research Institute Centre of Innovative Medicine, Vilnius, Lithuania; 3 Faculty of Mathematics and Informatics, Vilnius University, Vilnius, Lithuania

**Keywords:** Metabolic syndrome, Hypertriglyceridemia, Arterial stiffness, Endothelial dysfunction, Pulse wave velocity, Aortic augmentation index, Intima-media thickness

## Abstract

**Background:**

The development of metabolic syndrome (MS) augments risk for atherosclerotic cardiovascular disease (CVD), but pathophysiological mechanisms of this relation are still under discussion. Overlapping CVD risk factors make it difficult to assess the importance of individual elements. This study aimed to analyze subclinical atherosclerosis based on arterial structure and function parameters in patients with MS and different triglycerides levels.

**Methods:**

Patients (aged 40–65 years) were divided into two groups: patients with MS and with or without hypertriglyceridemia (hTG). Noninvasive assessment of vascular parameters—aortic augmentation index adjusted for heart rate 75 bpm (AIxHR75), pulse wave velocity (PWV), and common carotid artery intima-media thickness (cIMT) were performed.

**Results:**

Carotid-femoral PWV (cfPWV) and carotid-radial PWV (crPWV) were significantly higher in patients with hTG. After adjusting for age, gender, waist circumference, fasting glucose, smoking status, cardiovascular family history and mean arterial pressure, crPWV (OR 1.150; CI 95% 1.04–1.28), cfPWV (OR 1.283; CI 95% 1.14–1.42) and cIMT (OR 1.13; CI 95% 1.02–1.25) were significantly associated with hTG (p < 0.05), while AIxHR75 did not show significant association.

**Conclusion:**

Increased triglycerides are independently associated with a cfPWV, crPWV, and cIMT and may modify CVD risk in patients with MS.

**Supplementary Information:**

The online version contains supplementary material available at 10.1186/s12872-021-02202-3.

## Background

The metabolic syndrome (MS) is a cluster of obesity-associated cardiovascular risk factors including abdominal obesity, impaired glucose tolerance, hypertriglyceridemia (hTG), decreased high-density lipoprotein cholesterol (HDL-C), and increased blood pressure. Each component of the MS is an independent risk factor for cardiovascular disease (CVD) and the development of MS augments risk for type 2 diabetes mellitus and atherosclerotic CVD [1].

The discussion of MS remains to determine whether a single underlying pathophysiological substrate exists that consolidates the MS components or it involves multiple etiologic mechanisms. The pathophysiological mechanism by which the MS increases cardiovascular risk also remains under debate. In recent years the link between MS and atherosclerosis has been analyzed increasingly. Studies have shown that the increased grade of obesity and MS score are associated with accelerated atherosclerosis and a greater incidence of coronary heart disease [2]. Patients with MS have an approximately twofold increased risk for myocardial infarction [3]. It is often stated that insulin resistance is a basic component of MS [4]. Insulin resistance could promote atherosclerosis through mechanisms that involve systemic factors, such as dyslipidaemia, hypertension, and a proinflammatory state, also through the mechanisms at the cellular level [5, 6]. During the last decades, interleukin-1 (IL-1) family were significantly associated with the initiation and progression of obesity-induced insulin resistance [7]. High-sensitivity C-reactive protein (hsCRP) were associated with future insulin resistance in non-diabetic adults [8]. Clear evidence indicates that cytokines, for instance, adipokines, hepatokines, inflammatory cytokines (IL-1, IL-6), myokines, and osteokines, contribute substantially to the development of abnormal glucose and lipid metabolism [9].

Nevertheless, other components of MS, such as triglycerides (TG) also are important. There is no doubt that TG is a residual, modifiable, and causal atherosclerotic CVD risk factor [10]. However, the assessment of the relationship between TG and CVD remains controversial due to the coincidence of TG with other risk factors.

Therefore, the aim of the present study was to analyze subclinical atherosclerosis (for carotid artery intima-media thickness—cIMT) and arteriosclerosis (for pulse wave velocity—PWV) in high-risk patients with MS and different TG levels.

## Methods

All patients included in this community-based cross-sectional study were recruited from the Lithuanian High Cardiovascular Risk (LitHiR) primary prevention program. Methods are taken from LitHiR program methodology. A detailed description of the LitHir program protocol is presented elsewhere [11].

### Study design

The inclusion criteria were: men and women age between 40 and 65 years without overt cardiovascular disease (see exclusion criteria), diagnosed with MS, who were investigated between 2006 and 2015 years in Vilnius University Hospital Santaros klinikos. The study protocol has been priorly approved by the Lithuanian Bioethics Committee. Informed consent was waived by Lithuanian Bioethics Committee due to the retrospective study design.

The exclusion criteria were: (a) proved (clinically evident) coronary heart disease: myocardial infarction or unstable angina, angina on effort with positive stress test results, coronary pathology found on coronary angiography or multislice computed tomography angiography, coronary artery bypass grafting, or angioplasty, acute coronary events in the past; (b) proved (clinically evident) cerebrovascular disease: acute cerebrovascular events in the past or proved stenoses in the carotid arteries; (c) proved (clinically evident) peripheral artery disease: acute ischaemic syndrome, proved chronic ischaemia in limbs, aortic aneurism; (d) end-stage oncological disease; (e) any other end-stage somatic disease.

The MS was defined according to the revised National Cholesterol Education Program Adult Treatment Panel III (NCEP ATPIII) criteria, meeting three or more criteria: waist circumference ≥ 102 cm in men and ≥ 88 cm in women; systolic blood pressure (SBP) ≥ 130 mmHg and/or diastolic blood pressure (DBP) ≥ 85 mmHg, or if the diagnosis of hypertension was documented in the medical record; fasting plasma glucose ≥ 100 mg/dL or type 2 diabetes mellitus; TG concentration ≥ 150 mg/dL or special treatment is administered to reduce TG concentration; HDL-C < 40 mg/dL in men, < 50 mg/dL in women [12]. All patients were categorized into two groups: patients with MS and hTG (TG > 200 mg/dL) and a control group – patients without hTG (TG < 150 mg/dL).

### Study variables

All participants filled in the structured questionnaire about demographic and social characteristics, including age, cardiovascular family history, and smoking (see in Additional file 1). All the patients underwent measurements of height, weight, waist circumference, and arterial blood pressure. Waist circumference was measured midway between the top of the hip bone and the bottom of the ribs. Body mass index (BMI) was calculated as body weight (kg) divided by height (m) squared. Arterial hypertension was determined when SBP was ≥ 140 mmHg and/or DBP was ≥ 90 mmHg, or diagnosis of hypertension was documented in a medical record [13]. All the patient’s laboratory tests were performed in the morning after 12 h of fasting, and the following variables were determined: total cholesterol (TC), low-density lipoprotein cholesterol, (LDL-C), HDL-C, TG, and fasting glucose (FG).

### Non-invasive assessment of arterial stiffness

The participants were refrained from eating and drinking alcohol, coffee, or tea for at least 12 h before the study. The test of arterial stiffness was performed in the supine position in a quiet, temperature-controlled room (22–24 °C). PWV was determined by measuring the carotid-to-radial (crPWV) and carotid-to-femoral (cfPWV) pulse wave transit time. Carotid-radial or carotid-femoral pulse waves were obtained non-invasively by applanation tonometry using high-fidelity micromanometer (Sphygmocor (v.7.01) AtCor Medical Pty. Ltd 1999–2002, Sydney, Australia). Pulse waves obtained consecutively from the radial or femoral and carotid arteries were referenced to a simultaneously recorded ECG, and transit time was computed from the time difference between the carotid and radial or femoral waveforms. The distance between the surface markings of the sternal notch and the radial or femoral artery was used to estimate the difference in path length between the carotid and radial or femoral arteries, and PWV adjusted for mean blood pressure was calculated. Aortic augmentation index (AIx) adjusted for heart rate 75 bpm (AIxHR75) was calculated from radial pulse waves of the non-dominant arm [14, 15]. Validated transfer function from peripheral pulse wave analysis was used to generate a corresponding central waveform. From this, aortic AIx was calculated by using the integrated software. The systolic part of the central arterial waveform is characterized by two pressure peaks. The first peak is caused by left ventricular ejection, whereas the second peak is a result of pulse wave reflection. The difference between both pressure peaks reflects the degree to which central arterial pressure is augmented by wave reflection. AIx, a measure of systemic arterial stiffness, is calculated as the difference between the second and first systolic peaks expressed as a percentage of the pulse pressure [16]. Blood pressure was recorded in the left arm using an automatic blood pressure monitor (HEM-757; Omron Corporation, Kyoto, Japan).

### Carotid artery ultrasound examination

Carotid artery ultrasound examination was performed with high-resolution ArtLab Esaote (Italy) ultrasonic apparatus with an automatic Wall Tracing System, which consists of multiple sensors (Doppler signal in conjunction with a 128 Hz frequency radio wave signal). Before an examination, the patient lies on the back for 10 min. The sensor is placed on the outstretched neck and the optimal view of the carotid artery needs to be found. cIMT is measured in the common carotid artery 1–2 cm from the bifurcation on the bottom wall in a live two-dimensional view, which comprises 4 cm automatic recognition. The result is alive 6 s’ video clip together with ECG. Radiofrequency signal resolution is 0.21 μm.

### Statistical analysis

Data were presented as numbers and frequencies with percentages for categorical variables, and mean ± standard deviation (SD) for continuous variables. Group differences were tested using Student’s t-tests because sample sizes were sufficiently large and the assumption of normality could be made according to the central limit theorem [17]. Multivariable logistic regression was used to identify associations of arterial parameters and hTG after adjusting for age, gender, waist circumference, FG, smoking status, and cardiovascular family history. Each of the four main arterial parameters (cfPWV, crPWV, AIxHR75, and cIMT av.) were included separately in the models to avoid collinearity. In each case, three types of models were constructed, unadjusted (Model 1), adjusted only for age and gender (Model 2), and adjusted for major risk factors (Model 3). Overall, 12 (4 × 3) different models were tested.

A P value < 0.05 was considered statistically significant. All analyses were done using STATISTICA software (version 10).

## Results

A total of 1938 patients with MS were included in this study, 1041 of them had hTG (585 men, 52.24 ± 6.22 years). The basic characteristics of the study population are shown in Table 1. Significant differences were displayed in the levels of age, gender, weight, waist circumference, SBP and DBP, TC, LDL-C, DTL-C, FG, and smoking status between MS patients with hTG (> 200 mg/dL) and normal TG levels (< 150 mg/dL).

**Table 1:** Basic characteristics and MS components of the study population

Variable	MS + hTG (n = 1041)	Control group (n = 897)	p value
Age (years)	52.24 ± 6.22	54.63 ± 5.92	< 0.001
Women	456 (43.8%)	586 (65.3%)	< 0.001
Men	585 (56.2%)	311 (34.7%)	< 0.001
BMI (kg/m^2^)	31.99 ± 4.14	32.08 ± 4.22	0.648
BMI > 30 kg/m^2^	969 (66.9%)	622 (69.3%)	0.243
Weight (kg)	94.80 ± 15.91	91.20 ± 15.16	< 0.001
Waist circumference (cm)	107.66 ± 10.25	106.20 ± 10.01	0.002
SBP (mmHg)	142.01 ± 15.64	139.43 ± 15.83	< 0.001
DBP (mmHg)	87.72 ± 10.72	84.52 ± 11.13	< 0.001
MAP (mmHg)	106.05 ± 11.13	103.88 ± 10.91	< 0.001
FG (mg/dL)	115.14 ± 27.21	112.97 ± 17.30	0.045
Diabetes	217 (20.9%)	217 (24.2%)	0.078
TC (mg/dL)	269.53 ± 56.07	228.15 ± 46.79	< 0.001
LDL-C (mg/dL)	160.87 ± 50.66	155.84 ± 42.15	< 0.001
HDL-C (mg/dL)	41.37 ± 9.28	49.50 ± 12.37	< 0.001
TG (mg/dL)	333.91 ± 200.17	112.48 ± 24.80	< 0.001
Active smokers	290 (30.9%)	176 (19.6%)	< 0.001

MS, metabolic syndrome; hTG, hypertriglyceridemia; BMI, body mass index; SBP, systolic blood pressure; DBP, diastolic blood pressure; FG, fasting glucose; TC, total cholesterol; HDL-C, high-density lipoprotein cholesterol; LDL-C, low-density lipoprotein cholesterol; TG, triglycerides; MAP, mean arterial pressure

In hTG group the mean cfPWV was 8.74 ± 1.58 m/s, mean crPWV 9.24 ± 1.26 m/s, AIxHR75 21.21 ± 10.57, and average of cIMT was 666.36 ± 103.22 μm. As shown in Table 2, cfPWV, crPWV, and mean arterial pressure (MAP) were significantly higher in patients with hTG, while AIxHR75 was the lowest in this group. cIMT was the highest in the control group, but this finding was statistically insignificant. In a logistic regression analysis, both cfPWV and crPWV were significantly associated with hTG even in the model without adjustment for potentially influencing variables (Model 1). After adjusting for age, gender, waist circumference, FG, smoking status, cardiovascular family history and, MAP (Model 3), crPWV (OR 1.150; CI 95% 1.04–1.28), cfPWV (OR 1.283; CI 95% 1.14–1.42), and cIMT (OR 1.13; CI 95% 1.02–1.25) showed a significant association with hTG (p < 0.05). Similar associations were found after adjusting only for age and gender (Model 2). In the nonadjusted model (Model 1), AIxHR75 showed reversed association, but in adjusted models, the results were not significant. (Table 3).

**Table 2:** Arterial structure and function parameters in study groups

Variable	MS + hTG (n = 1041)	Control group (n = 897)	p value
cfPWV (m/s)	8.74 ± 1.58	8.51 ± 1.6	< 0.002
crPWV (m/s)	9.24 ± 1.26	8.91 ± 1.28	< 0.001
AIxHR75	21.21 ± 10.57	23.44 ± 10.01	< 0.001
cIMT (μm)	666.36 ± 103.22	671.74 ± 101.94	0.25

MS, metabolic syndrome; hTG, hypertriglyceridemia; cfPWV, carotid-femoral pulse wave velocity; crPWV, carotid-radial pulse wave velocity; AIxHR75, aortic augmentation index adjusted for a heart rate 75 bpm; cIMT, an average of common carotid artery intima-media thickness

**Table 3:** Association between vascular structure and function parameters and hTG in patients with MS^a^

Variable	Model 1		Model 2		Model 3	
	OR (95% CI)	p	OR (95% CI)	p	OR (95% CI)	p
cfPWV	1.16 (1.05–1.26)	0.002	1.30 (1.18–1.43)	< 0.001	1.28 (1.14–1.42)	< 0.001
crPWV	1.31 (1.19–1.33)	< 0.001	1.21 (1.10–1.33)	< 0.001	1.15 (1.04–1.28)	0.007
AIxHR75	0.8 (0.73–0.88)	< 0.001	1.04 (0.93–1.16)	0.511	0.93 (0.82–1.05)	0.225
cIMT	0.95 (0.87–1.04)	0.486	1.11 (1.01–1.23)	0.037	1.13 (1.02–1.25)	0.021

Odds ratios were for a 1-standard deviation increase in continuous parameters

OR, odds ratio; cfPWV, carotid-femoral pulse wave velocity; crPWV, carotid-radial pulse wave velocity; AIxHR75, aortic augmentation index adjusted for a heart rate 75 bpm; cIMT, an average of common carotid artery intima-media thickness

^a^Model 1: nonadjusted; Model 2: adjusted for age and gender; Model 3: adjusted for age, gender, waist circumference, fasting glucose, smoking status, cardiovascular family history, and MAP

## Discussion

The main finding of the present study is that an increased TG was independently associated with a cfPWV, crPWV, and cIMT, but not with higher AIxHR75. PWV is a useful biomarker for the prediction of CVD risk [18]. It correlates well not only with the presence of coronary, cerebral, and carotid artery atherosclerosis but also with its extent [19]. An association between hTG and PWV has been shown previously [20–22]. The higher level of TG was demonstrated as an independent predictor of increased cfPWV [23]. The current study combines previous reports and shows that hTG could impact PWV in patients with MS. In our study, both crPWV and cfPWV were associated with hTG after adjusting for mean blood pressure, age, gender, waist circumference, FG, and more potentially influencing variables.

The relationship between TG and PWV could be mediated through left ventricular mass (LVM). TG is associated with LVM index in hypertensive patients, independently of other risk factors [24]. Whereas vascular stiffness induce left ventricular hypertrophy, PWV is a surrogate marker for LVM. To further enhance the predictive role of TGs, a recent study showed that TG and remnant cholesterol, but not LDL cholesterol, are associated with CVD outcomes [25]. AixHR75, as well as PWV, is an early marker of vascular changes in stiffness and elasticity and more useful in younger subjects. Although there is controversy about the prognostic role of Aix [26], a meta-analysis demonstrated that AIx75 is an independent predictor of future cardiovascular events and all-cause mortality [27]. The current study showed a disparity in aortic AIxHR75—the patients with lower TG levels had higher AIxHR75, but it disappeared after adjusted influencing variables. Obesity may be the key factor that mediates this relationship. In our study, more obese patients were in a control group, and their BMI was higher, though waist circumference was smaller. Although some studies are emphasizing central obesity [28], a meta-analysis confirmed the relationship between obesity/overweight and increased AIX without underestimating general obesity [29]. Moreover, increased pro-inflammatory factors (IL-6, TNF-α) may influence increased arterial stiffness [30, 31], while in our study they were not analyzed.

Several studies have shown an association between cIMT and future CVD events [32]. A longitudinal, 13 years follow-up study showed MS association with progression of cIMT, but only in those below 50 years of age [33]. Several studies have found that all single components of the MS may promote endothelial dysfunction leading to arterial injury. We also find an association between cIMT and TG level which appeared after adjusting for influencing variables.

Well-known factors leading to arterial stiffening are arterial hypertension, diabetes, and chronic kidney diseases. However, new promising research directions are mitochondrial oxidative stress, genome mutations, epigenetics, metabolism, and dyslipidemia [34]. Despite the fact that the correlation between fasting TG levels and cIMT remains controversial [35], there is growing evidence about hTG impact on oxidative stress and inflammation [36] which leads to the thickening of artery walls and atherosclerosis [23]. Limitations of the current analysis include the design and criteria of a preventive program. We could not carry out analyses of certain age groups and perform a follow-up. Moreover, the study includes only a Lithuanian population and the results cannot be directly applied to people of other nationalities. The strengths of this study include its large sample from a community-based population and adjustment for many covariates.

## Conclusions

Higher TG is associated with cfPWV, crPWV, and cIMT but not with the AixHR75 in patients with MS. Whereas vascular changes are associated with an increased CVD risk, high TG may differentiate the prognosis of patients with MS.

## Supplementary Information

**Additional file 1**. Study questionaire.

## Data Availability

The datasets generated and analyzed during the current study are not publicly available due to the fact that the LitHiR program is continued and the data is filled, but are available from the corresponding author on reasonable request.

## Abbreviations

AIX: Aortic augmentation index
AIxHR75: Aortic augmentation index adjusted for heart rate 75 bpm
BMI: Body mass index
CCA IMT: Common carotid artery intima-media thickness
cfPWV: Carotid-femoral PWV
crPWV: Carotid-radial PWV
CVD: Cardiovascular disease
DBP: Diastolic blood pressure
FG: Fasting glucose
HDL-C: High-density lipoprotein cholesterol
hTG: Hypertriglyceridemia
IMT: Intima-media thickness
LDL-C: Low-density lipoprotein cholesterol
MAP: Mean arterial pressure
MS: Metabolic syndrome
OR: Odds ratio
PWV: Pulse wave velocity
SBP: Systolic blood pressure
SD: Standard deviation
TC: Total cholesterol
TG: Triglycerides
