# Supplementary material for: Hypertriglyceridemia impact on arterial parameters in patients with metabolic syndrome
Source: BMC Cardiovasc Disord. 2021 Aug 13;21:393. doi: 10.1186/s12872-021-02202-3 (PMC8361842; doi:10.1186/s12872-021-02202-3)
Supplement: Supplementary file 1 — Additional file 1. Study questionaire. [file 12872_2021_2202_MOESM1_ESM.docx]

**Hypertriglyceridemia impact on arterial parameters in patients with metabolic syndrome**

Egidija Rinkūnienė1*, Vilma Dženkevičiūtė1, Žaneta Petrulionienė1, Eglė Majauskienė1, Ligita Ryliškytė1, Roma Puronaitė1, 3, Jolita Badarienė1, Rokas Navickas1, Aleksandras Laucevičius1,2.

1 Faculty of Medicine, Vilnius University, Vilnius, Lithuania

2 State Research Institute Centre of Innovative Medicine, Vilnius, Lithuania

3 Faculty of Mathematics and Informatics, Vilnius University, Vilnius, Lithuania

*Corresponding author: egle.majauskienee@gmail.com

Vilnius University Hospital Santaros Klinikos, Vilnius, Lithuania.

Santariskiu Str. 2, Vilnius, 08661, Lithuania.

| **Study questionaire** |  |
| --- | --- |
| 1. Age 2. Gender | \| years \| \| --- \| \|  \| |
| 1. Cardiovascular family history:   a) First degree relatives with Cardiovascular Disease (high blood pressure, heart attack, angina, stroke, hardening of the arteries) at a young age (women less than 65 years or man less than 55 years)  b) First degree relatives with diabetes | \| Yes \| No \| \| --- \| --- \|  \| Yes \| No \| \| --- \| --- \| |
| 1. Physical activity: 2. Physical activity less than once a week 3. Physical activity 2-3 times per week 4. Physical activity 4-5 times per week 5. Physical activity 5 or more times per week | \| Yes \| \| --- \| \| Yes \| \| Yes \| \| Yes \| |
| 1. Smoking: 2. Never smoked 3. Ex-smoker   i) duration of smoking   1. Current smoker less than 10 cigarettes/day 2. Current smoker more than 10 cigarettes/day | \| Yes \| No \| \| --- \| --- \| \| Yes \| No \| \| years \| \| \| Yes \| No \| \| Yes \| No \| |
| 1. Personal history of arterial hypertension 2. Diagnosed 3. Treated with medication | \| Yes \| No \| \| --- \| --- \| \| Yes \| No \| |
| 1. Personal history of diabetes 2. Diagnosed 3. Treated with oral medication 4. Treated with insulins | \| Yes \| No \| \| --- \| --- \| \| Yes \| No \| \| Yes \| No \| |
|  |  |
